# Dynamic Functional Connectivity Markers in Anorexia Nervosa and Their Association With Clinical Symptoms: A Cross‐Sectional Study

**DOI:** 10.1002/erv.3188

**Published:** 2025-03-10

**Authors:** Feliberto de la Cruz, Monica Di Giuliano, Katrin Rieger, Andy Schumann, Karl‐Jürgen Bär

**Affiliations:** ^1^ Lab for Autonomic Neuroscience Imaging and Cognition (LANIC) Department of Psychosomatic Medicine and Psychotherapy Jena University Hospital Jena Germany

**Keywords:** anorexia nervosa, body image disturbance, brain states, dynamic functional connectivity, somatomotor network, ventral attentional network

## Abstract

The human brain possesses a unique ability to switch between patterns of functional connectivity, known as brain states, which are crucial for regulating biological, cognitive, and emotional processes. These states are linked to numerous neurological and neuropsychiatric conditions, however, their relationship to clinical symptoms of anorexia nervosa (AN) is not well understood. In this exploratory study, we aimed to identify whole‐brain dynamic functional alterations in AN and their association with AN symptoms. To this end, we recruited 19 women diagnosed with AN and 22 healthy controls (HCs) who underwent resting‐state functional magnetic resonance imaging. By implementing a sliding‐window approach, we found that AN patients had limited flexibility to transit between different brain configurations compared to HCs. Moreover, AN patients spent a significant amount of time in a functional configuration characterised by strong coupling between the ventral attentional network and the somatomotor network. Changes in these networks play a crucial role in body image disturbances, interoceptive awareness, and body image‐body schema interaction. Interestingly, the time spent in this specific brain state showed a negative relationship with body mass index, along with a positive correlation with eating disorder indices. Our study highlights the potential of dynamic functional connectivity as a prognostic and therapeutic means to spotlight maladaptive functional brain configurations in AN.

**Summary**

- AN patients showed less plastic flexibility to transit between different brain states compared to HCs.
- We identified a clinically relevant brain state in which AN patients spent nearly half of their time, characterised by strong coupling between the ventral attentional and somatomotor networks.
- This clinically relevant brain state correlated with body mass index and positively with eating disorder scale indices.
- Overall, dynamic functional connectivity offers valuable insights into potential diagnostic and therapeutic markers for AN.

## 1 Introduction

Anorexia nervosa (AN) is a severe mental disorder that predominantly affects adolescent girls and young women, with the highest mortality rate among all mental health disorders (Papadopoulos et al. 2009; Arcelus et al. 2011). Characterised by restrictive eating behaviours, an overwhelming fear of weight gain, and significant distortions in body image perception, AN encompasses a complex array of psychological and physiological manifestations (Zhong et al. 2023). The principal diagnostic criterion for AN is markedly low body weight in relation to an individual's height, age, developmental stage, or weight history, as defined in the International Classification of Diseases (ICD‐11). This clinical profile is often accompanied by psychological traits such as perfectionism, excessive physical activity, and alexithymia (Lilenfeld et al. 2006). Despite advances in research, no proven treatment currently exists to effectively normalise the core symptoms of AN, highlighting the need for more in‐depth investigations into the underlying neuropsychological mechanisms (Kaye et al. 2020).

A substantial body of research indicates that the clinical features of AN are sustained by disruptions within an extensive neural network that includes the limbic system, prefrontal cortex, temporal gyri, insula‐opercular cortex, and cerebellum (Wagner et al. 2003; Via et al. 2018; Zhong et al. 2023; Alfano et al. 2020; Q. Zheng et al. 2024; Seeger et al. 2002; Sachdev et al. 2008; Karakuş Aydos et al. 2023). Notably, abnormalities within the frontoparietal network and the anterior and posterior nodes of the Default Mode Network (DMN) are associated with alterations in attentional, visuospatial, and self‐referential mechanisms relevant to body image in AN (Wagner et al. 2003; Via et al. 2018). Increased activation of parietal regions is implicated in visuospatial processing, attentional regulation, and self‐awareness, whereas extensive temporal lobe activity correlates with body dissatisfaction and emotional avoidance strategies (Alfano et al. 2020; Q. Zheng et al. 2024). The limbic system, alongside the claustrum, is integral to emotional regulation, interoceptive awareness, sensorimotor integration, and memory processing (Seeger et al. 2002). Furthermore, the cerebellum—particularly its anterior and posterior areas—plays a crucial role in the distorted body image characteristic of individuals with AN (Sachdev et al. 2008; Karakuş Aydos et al. 2023).

The investigation of brain signal dynamics is crucial for elucidating the mechanisms that underlie cognitive, behavioural, and emotional processes in neuropsychiatric disorders (Zagha and McCormick 2014; Ramirez‐Mahaluf et al. 2020; Cabral et al. 2017; Hutchison et al. 2013). In this context, functional connectivity (FC) serves as a key parameter for assessing spontaneous low‐frequency oscillations and quantifying the synchronisation of neural activity among distinct brain regions (Friston 2011). However, the standard static FC approach only yields a single connectivity index, which limits our ability to detect dynamic brain changes that characterise complex mental illnesses (Damaraju et al. 2014; Peng et al. 2023). To overcome this limitation, monitoring changes in FC over time can be an effective strategy. Fluctuations in FC may reflect dynamic alterations in the macro‐scale spatiotemporal organisation of brain networks, which are associated with shifts in human behaviour and cognition, as well as various neurological conditions (Shakil et al. 2016; Xia et al. 2019). Within this narrative, the concept of “brain states” emerges as crucial to understanding brain dynamics. Brain states refer to recurrent neural conditions stable for behaviourally significant periods (Zagha and McCormick 2014). These states represent reliable patterns of brain activity involving the co‐activation and/or connectivity of multiple large‐scale brain networks, with transitions between states vital for self‐regulation and adaptation to varying environments (Tang et al. 2012). The temporal properties of brain states, including the duration spent in specific states and the flexibility of transitions, bear biological significance. For instance, reduced transitions between brain states correlate with diminished cognitive abilities, while an extended duration in energetically costly states can hinder adaptive functioning during cognitively demanding tasks (Ramirez‐Mahaluf et al. 2020; Cabral et al. 2017).

In neurological and psychiatric disorders, analysing brain states can reveal disease‐specific patterns. For example, transient dysconnectivity states are characteristic of schizophrenia, while pathological dwell times are observed in major depressive disorder (Damaraju et al. 2014; Holtzheimer and Mayberg 2011). However, despite its extensive applications, the potential of dynamic FC analysis remains largely unexplored in AN with only two published studies to date (De la Cruz et al. 2023; Boehm et al. 2024). One of these studies revealed that the neurocircuitry underlying autonomic cardiac dysfunction in AN transits across five functional states, with heart rate variability differing between healthy controls and AN patients at the state of weakest connectivity. The study also found a linear relationship between the time spent in this state and body mass index. However, this study focused on only a few seed regions, indicating the need for further research into whole‐brain dynamic alterations in AN (De la Cruz et al. 2023). The second study investigating resting‐state dynamics in AN revealed that individuals with AN spent little time in a weakly connected state and had reduced transitions between states (Boehm et al. 2024). An intriguing finding from this study was the absence of a relationship between dynamic FC measures and various clinical and demographic variables, such as body mass index or scores related to eating disorders.

Thus, this exploratory study aimed to identify whole‐brain dynamic FC differences in AN. Using a sliding‐window approach, we derived functional brain states and hypothesised that AN patients would exhibit fewer state transitions, longer dwell times, and increased time in specific brain states compared to healthy controls (HCs). Additionally, we explored potential associations between dynamic FC measures and clinical characteristics such as BMI, sociodemographic features, and clinical scale scores.

## 2 Materials and Method

### 2.1 Participants

We recruited 19 female patients with AN and 22 age and gender‐matched HCs. All patients met the DSM‐IV criteria for AN according to the Structured Clinical Interview for DSM‐IV Axis I disorders (SCID‐I; First et al. 2002). To assess general psychopathology, we utilised a set of self‐report questionnaires including the Eating Disorder Inventory‐2 (EDI‐2; Thiel et al. 1997), the State–Trait Anxiety Inventory (STAI trait, STAI state; Laux 1981), the Beck Depression Inventory (BDI‐2) self‐report questionnaire (Beck et al. 1996), the Behavioural Inhibition Questionnaire (BIS; Carver and White 1994), the Trail Making Test, version A and B (TMT‐A/B; Reitan 1955), the Toronto Alexithymia Scale (TAS‐26; Bagby et al. 1994), the Affective Neuroscience Personality Scales (ANPS; Davis et al. 2003), the Urgency, Premeditation, Perseverance, and Sensation seeking Impulsive Behaviour Scale (UPPS; Whiteside and Lynam 2001), and the Mehrfachwahl‐Wortschatz‐ Intelligenztest (MWT‐B; Lehrl 2005). The main clinical and amnestic data are summarised in Table 1.

**TABLE 1:** Demographics and clinical data. Mean ± standard deviation values and range are reported for demographic and clinical parameters.

General	AN patients	Controls	Statistic^a^	*p* value
Age (years)	23.6 ± 5.0 (18–37)	22.6 ± 1.9 (18–25)	*t* = 0.94	0.35
BMI (kg/m)	15.7 ± 1.3 (13.3–17.8)	21.9 ± 1.7 (19.2–24.8)	*t* = 12.8	< 0.001
Eating disorder history and scales				
EDI‐2 total score	326.3 ± 47.0 (216–414)	200.4 ± 37.7 (120–297)	*t* = 9.63	< 0.001
STAI trait	47.9 ± 6.2 (36–64)	45.4 ± 2.7 (39–52)	*t* = 1.74	0.09
STAI state	46.4 ± 11.6 (32–67)	33.7 ± 4.9 (26–45)	*U* = 375.5	< 0.001
BDI‐2 score	28.7 ± 12.8 (4–57)	4.7 ± 5.0 (0–19)	*t* = 8.21	< 0.001
TMT‐a	22.5 ± 8.7 (11.8–49.7)^b^	26.9 ± 6.9 (16.6–41.7)	*t* = 1.80	0.08
TMT‐b	53 ± 14.7 (31.1–80.9)^c^	51.6 ± 15.6 (28.7–85.9)	*t* = 0.29	0.78
BIS	55.7 ± 13.0 (44–90)	56 ± 8.5 (45–83)	*t* = 0.06	0.95
TAS‐26	53.3 ± 11.3 (34–69)	42 ± 9.5 (29–67)	*t* = 3.50	< 0.001
ANPS	269.8 ± 15.9 (248–312)^d^	273.1 ± 20.1 (222–298)	*t* = 0.56	0.58
UPPS	97.1 ± 16.4 (70–130)^e^	99 ± 15.3 (71–132)	*t* = 0.37	0.72
MWTB	28.4 ± 3.2 (23–33)	29.2 ± 4.8 (16–37)	*t* = 0.64	0.52
Education				
No	*n* = 0	*n* = 0	*χ* ^2^ = 5.81	0.02
Primary	*n* = 0	*n* = 0		
Secondary	*n* = 6	*n* = 0		
Higher level	*n* = 13	*n* = 22		
N/A	*n* = 0	*n* = 0		
Physical activity (hours per week)				
From 1 to 2 h	*n* = 5	*n* = 9		
From 3 to 4 h	*n* = 3	*n* = 8		
From 5 to 6 h	*n* = 3	*n* = 0		
More > 6 h	*n* = 1	*n* = 2		
N/A	*n* = 7	*n* = 3		
Last menstrual cycle period				
Same year of scanning	*n* = 7	*n* = 17		
1 year before scanning	*n* = 3	*n* = 0		
2 years before scanning	*n* = 1	*n* = 0		
3 years before scanning	*n* = 1	*n* = 0		
> 3 years before scanning	*n* = 1	*n* = 0		
N/A	*n* = 6	*n* = 5		

Abbreviations: ANPS, affective neuroscience personality scales; BDI‐2, beck depression inventory 2; BMI, body mass index; BIS, behavioural inhibition questionnaire; EDI‐2, eating disorder inventory‐2; MWTB, Mehrfachwahl‐Wortschatz‐Intelligenztest; N/A, not accessible data; STAI, state‐trait anxiety inventory; TAS‐26, Toronto Alexithymia Scale.

^a^ The distribution of all variables was checked using the Kolmogorov‐Smirnov test, and group comparisons were performed using either *t*‐test or Mann‐Whitney *U*‐test, as appropriate.

^b^ Available in AN = 18.

^c^ Available in AN = 18.

^d^ Available in AN = 16.

^e^ Available in AN = 18.

Each participant underwent a thorough clinical examination and a screening for other disorders such as major depression, personality disorders, or obsessive‐compulsive disorder. The SCID‐I interview revealed that none of the HCs had a current episode or history of mental disorder. For AN, a medical expert confirmed from the patients' records that six AN patients had a depressive clinical profile in their medical history, one patient had social phobia disorder, and three had bulimia nervosa disorder in comorbidity with anorexia. The other AN patients had no relevant clinical diagnoses besides AN disorder. Seven patients were taking antidepressant medication, and six were taking antipsychotic medication during the study. Out of the 41 participants, 36 were right‐handed and five were left‐handed. There was incomplete demographic and clinical data for some participants due to variability in documentation and participants' refusal to provide information. All participants provided informed written consent, and the study was conducted with the approval of the local Ethics Committee of the University Hospital Jena and in accordance with the guidelines of the Helsinki Declaration (from 2013).

### 2.2 MRI Data Acquisition

MRI data were collected on a 3T whole‐body system equipped with a 64‐element head matrix coil (MAGNETOM Prisma, Siemens Healthcare, Erlangen, Germany). During scanning, participants were instructed to keep their heads still and relax with their eyes closed without falling asleep or having systematic thought. We acquired T2*‐weighted images using a multiband multislice GE‐EPI sequence with the following parameters: repetition time (TR) = 961 ms, echo time (TE) = 30 ms, flip angle (FA) = 90°, multiband factor = 6, matrix size = 98 × 98 with in‐plane resolution 2 × 2 mm^2^ and 72 contiguous transversal slices of 2 mm thickness covering the entire brain. The session lasted approximately 12 min and contained a series of 750 whole‐brain volume sets. In addition to functional data, we also acquired high‐resolution anatomical T1‐weighted volume scans (MPRAGE) in sagittal orientation. MP‐RAGE parameters were: TR = 2400 ms, TE = 2.34 ms, inversion time (TI) = 1.04 ms, FA = 8°, matrix size = 256 × 320, voxel size = 0,7 × 0,7 × 0,7 mm^3.^ The total number of slices was 320.

### 2.3 rsfMRI Preprocessing

We utilised the ‘afni_proc.py’ script from the AFNI software package (Cox 1996) to preprocess the rsfMRI data. After discarding the first five volumes, the rsfMRI data underwent despiking, motion correction, alignment to the anatomical scan, warping to the Montreal Neurological Institute template, and smoothing with a 6‐mm full‐width half‐maximum Gaussian kernel. We then applied a filter to retain frequencies in the 0.01–0.1 Hz frequency band and reduced non‐neural sources by linear regressing motion parameters and three principal components of ventricle signals. The ventricle mask was generated from each participant's anatomical scan using Freesurfer 7.4.1 (http://surfer.nmr.mgh.harvard.edu). It is worth noting that we did not regress the white matter signal, as current literature suggests it may provide important spatiotemporal information regarding the functional architecture of the whole brain, similar to grey matter (P. Wang et al. 2022).

### 2.4 Dynamic FC

We utilised a sliding‐window approach to estimate temporal changes in FC. Our analysis involved splitting the time series into windows of 60‐s length, with the onset of each window progressively shifted by 20 s (40 TR) from the previous window. This resulted in a total of 35 windows. Our window size of 60 s and overlapping of 40 s results in a good balance between the sensitivity of detecting potentially interesting transients in FC and the signal‐to‐noise ratio of the estimated FC (Deco et al. 2017). We calculated dynamic FC matrices for each window by computing Pearson correlation coefficients between 300 regions. To achieve this, we utilised the well‐established Schaefer parcellation model (Schaefer et al. 2018). From the extensive range of parcellation schemas available in this model, we selected 300 parcels and extracted their average time series. Connectivity patterns identified at this level of granularity are particularly effective for capturing individual differences in behaviour and cognitive function (Wu et al. 2021)⁠. We then concatenated all dynamic FC matrices across all participants and identified the optimal number of clusters (kernels) using the elbow method. This involved performing K‐means clustering with varying numbers of *k* (2–14 in this study) and identifying the ‘elbow’ point where increasing *k* yields minimal gains in the sum of squared distances. This consistently resulted in five cluster centroids or brain states, which we used to classify the dynamic FC matrices for each subject via k‐means clustering with the Euclidean distance as the cost function. Next, we estimated three brain state parameters to compare dynamic FC between AN and HCs and explore potential associations with clinical variables. These parameters included: the total cumulative duration that a participant remains in a specific brain state across the entire scan (time spent); the average time a participant stays in a certain state before changing to another state (dwell time), and the probability of transition from one state to another (flexibility index).

### 2.5 Statistical Analysis

We performed statistical analyses using the Python SciPy package. First, all variables were tested for normality using the Kolmogorov–Smirnov test. Based on these results, we compared demographic and clinical characteristics (e.g., gender, age, EDI‐2) as well as dynamic features (time spent, dwell time, and flexibility index) using *t*‐tests, chi‐square tests, or Mann–Whitney U‐tests, as appropriate. We also assessed relationships between variables using Pearson or Spearman correlations, depending on the distribution of the data. Given the exploratory nature of the correlation analysis, we did not apply a correction for multiple comparisons to the *p*‐values. However, for group comparisons on dynamic features (time spent and dwell time), we applied a Bonferroni correction by multiplying the *p*‐value by the number of brain states. The results were expressed in Cohen's *d* values, with Hedge's *g* correction applied for small sample sizes.

## 3 Results

**FIGURE 1:**
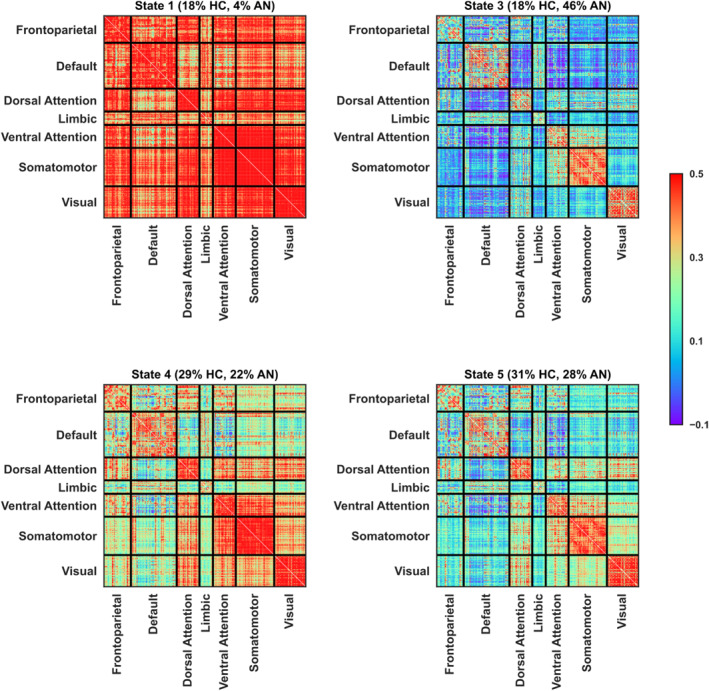
K‐means clustering identified 5 brain functional states in both healthy individuals and individuals with anorexia nervosa, with state 2 present in only 1 AN patient and not shown here. Brain states are represented as connectivity matrices and organised by functional networks. Positive correlations are depicted in red, while negative correlations are shown in blue. State 3 appears to be clinically relevant in anorexia nervosa. Patients spent nearly 46% of their time in this state, compared to the 18% observed in healthy individuals. State 3 is characterised by a strong coupling between the ventral attention and somatomotor networks while exhibiting weak functional connectivity with the default mode network and limbic systems. The time spent across states in healthy controls sums to 96%, not 100% because State 2 is not displayed; the single participant in whom this state was present accounted for the remaining 4%.

**FIGURE 2:**
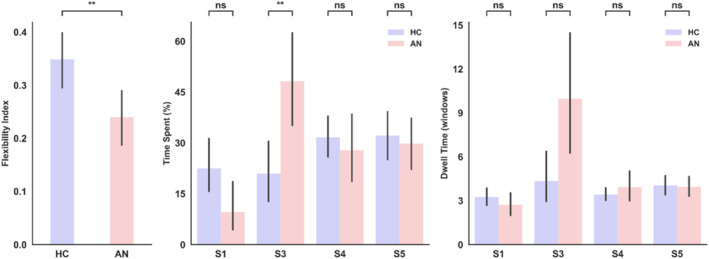
Group comparisons of dynamic features (flexibility index, time spent, and dwell time). **Left:** The total number of temporal transitions between brain states was significantly lower in AN patients compared to HCs (*p* = 0.008, Cohen's *d* = 0.85), indicating reduced plasticity in switching brain states. **Middle:** AN patients spent significantly more time in state 3 than HCs (Bonferroni‐corrected *p* = 0.012, Cohen's *d* = 1.04), while the time spent in other states did not differ significantly. **Right:** No significant group differences were observed in dwell time, though a trend was noted in state 3 (uncorrected *p* = 0.038, Bonferroni‐corrected *p* = 0.195, Cohen's *d* = 0.76), with AN patients remaining in state 3 for approximately five consecutive windows, compared to four in HCs. All differences between AN and HCs were tested using independent two‐sample *t*‐tests with Bonferroni correction.

The k‐mean clustering algorithm identified five brain functional states in both HCs and individuals with AN, with state 2 appearing in only one AN patient and therefore not further included as clinically and statistically relevant. Among these states, state 3 emerges as particularly clinically relevant in the context of AN, as shown in Figure 1. Notably, the time spent in this state differed significantly (Bonferroni corrected *p* = 0.012, Cohen's *d* = 1.04) between AN patients and HCs, with AN patients spending approximately 46% of their time in state 3. State 3 exhibited a strong coupling between the ventral attention (VAN) and somatomotor (SMN) networks, along with a weak coupling between the dorsal attention network and default mode and limbic networks. Further analysis indicated that AN patients are significantly (*p* = 0.008, Cohen's *d* = 0.85) less flexible when switching between states compared to HCs, as evidenced by their reduced flexibility index. Although not statistically significant (uncorrected *p* = 0.038, Bonferroni corrected *p* = 0.195, Cohen's *d* = 0.76), AN patients tended to dwell approximately five consecutive windows in state 3, compared to four for HCs, as shown in Figure 2.

### 3.1 Association of Dynamic Features With Clinical Variables

**FIGURE 3:**
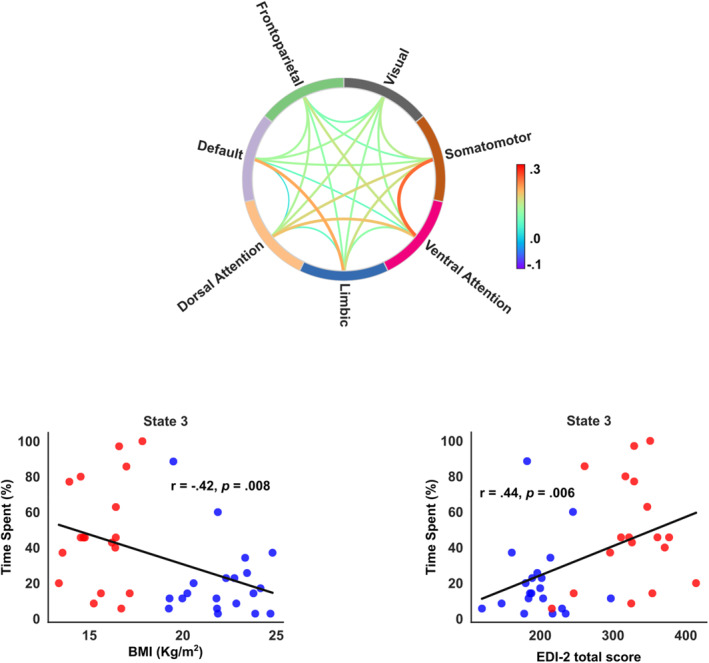
The chord diagram provides a simplified representation of the average functional coupling between networks in the clinically relevant state 3. Notably, it highlights the strong coupling between the ventral attentional and somatomotor networks, alongside the weaker coupling between other relevant networks such as the dorsal attention and default mode networks. Additionally, the association of the percentage of time spent in this clinically relevant brain state is depicted (red = patients; blue = HCs) with body mass index (uncorrected *p* = 0.008) and the total score in the EDI‐2 questionnaire (uncorrected *p* = 0.006).

We observed a significant negative correlation (uncorrected *p* = 0.008) between BMI and time spent in State 3 across groups, indicating that individuals with more severe symptomatology tended to spend more time in this state. Eating disorder symptoms were also positively correlated with the time spent in state 3 (uncorrected *p* = 0.006), as measured by the EDI‐2 score (Figure 3). Weak uncorrected correlations were also observed between the flexibility index and BMI and EDI‐II, as well as between time spent in state 3 and other clinical assessments (BDI, TAS‐26), as detailed in the Supplementary Material.

## 4 Discussion

This study investigated whole‐brain dFC alterations in AN patients. Our findings indicate that AN patients tend to remain in a functional configuration characterised by strong coupling between the VAN and SMN. This brain state appears to have clinical significance, as the duration spent in this configuration correlates with symptoms such as BMI and EDI‐2 total scores. In line with their prolonged time in this potentially clinically relevant state, AN patients also exhibited reduced flexibility in transitioning between different brain states, which may reflect the cognitive inflexibility often observed in this disorder.

Our analysis of brain states using dFC analysis revealed a potentially clinically relevant state for patients suffering from AN. Specifically, we found that AN patients spent approximately 46% of their time in state 3, whereas healthy controls only spent 18% of their time in this state. This increased proportion of time in a specific brain configuration likely reflects lower cognitive flexibility (Ehrlich, Geisler, et al. 2015). Research shows that low cognitive flexibility is a key trait in AN, predicting disease onset (Treasure and Schmidt 2013; Ehrlich, Lord, et al. 2015) and contributing to traits such as elevated levels of perfectionism and obsessive‐compulsive behaviours (Treasure and Schmidt 2013; Ehrlich, Lord, et al. 2015). Spending more time in a particular brain state may thus act as a neural correlate of these core AN traits and serve as a potential biomarker for monitoring disease progression.

Regarding dwell time, recent studies suggest that shorter dwell times may reflect more efficient cognitive control, while longer dwell times suggest cognitive inflexibility (Hutchison and Morton 2015; Kupis et al. 2021). In line with this, we observed that AN participants tend to exhibit longer dwell times in the clinically relevant brain state compared to healthy controls. Similarly, our analysis revealed a significant difference in the flexibility index between AN and healthy controls. AN patients showed a reduced capacity to switch between states, indicating a tendency to become “stuck” in a particular state, further suggesting cognitive rigidity. Moreover, a reduced flexibility index in AN is consistent with lower performance on behavioural measures of executive function often seen in AN individuals (Nomi et al. 2017; Kupis et al. 2021). Abnormal flexibility can also be understood within the concept of metastability. Current research suggests that neural activity depends on the ability to rapidly coordinate and flexibly orchestrate neural ensembles, avoiding becoming “locked” into fixed brain states (Friston 1997; Shanahan 2010; Hellyer et al. 2015). In the context of AN, the inability to dynamically switch between states could be an indicator of lower metastability and less efficient coordination of neural ensembles. This reduced metastability is particularly significant in the context of functional connectivity, as lower flexibility may result in impaired information transfer within and between large‐scale functional networks (J. Wang et al. 2019; R. Zheng et al. 2022). Overall, the observed patterns of longer dwell times, increased time spent in specific states, and lower flexibility indices further confirm that individuals with AN exhibit less adaptable cognitive processes.

One interesting characteristic of the potential clinically relevant brain state 3 is the strong coupling between the VAN and the SMN. This connectivity profile aligns well with the functions associated with both networks. The VAN plays a critical role in anorexia nervosa (AN), as evidence suggests it encodes body‐related stimuli and differentiates them from non‐bodily stimuli, with impairments in these processes being core traits of anorexia psychopathology (Kaye et al. 2009; O’Hara et al. 2015). Similarly, the SMN is central to research on body size evaluation, body image distortion, anosognosia, and elevated pain thresholds in AN (Nunn et al. 2008). SMN integrates visual and somatosensory information to support accurate body image perception, a process that often fails in AN, particularly when processing internal and external bodily states (Favaro et al. 2012; Esposito et al. 2018; Gaudio et al. 2018). Research reveals that SMN activity shifts with illness state, decreasing during acute AN and increasing in weight‐recovered individuals (Favaro et al. 2012; Phillipou et al. 2016).

Another significant finding in this study was the association between the fraction of time spent in state 3 with important clinical parameters relevant to therapeutic contexts. Specifically, we found a negative correlation between the percentage of time spent in this brain state with BMI, as well as a positive correlation with EDI‐2 total score. These findings are particularly significant because BMI is a crucial predictor of the disease's progression and response to treatment. Additionally, specific factors from the EDI total score—such as perfectionism, ineffectiveness, interpersonal distrust, interoceptive awareness, and drive for thinness—are well‐established prognostic indicators to consider in the early stages of the disorder (Bizeul et al. 2001; Bodell et al. 2016). Although it is the first study identifying associations between dynamic features and clinical parameters in AN, a recent study by Spalatro et al. (2019) found links between activity in the VAN, a relevant network in our clinically significant state 3, and various psychopathological measures, including BMI, TAS, and common eating disorder symptoms such as physical hyperintensity, drive for thinness, and impulsivity.

In contrast to other resting‐state fMRI studies, our findings did not reveal an abnormal hyperactivation of the dorsal attentional network or the default mode network (Boehm et al. 2014; Via et al. 2018; Gondo et al. 2023). This discrepancy may stem from our different methodological approach; we examined whole‐brain functional couplings between major predefined seed networks rather than using an independent component analysis, which is a data‐driven approach for identifying components of interest (Boehm et al. 2024). However, consistent with Boehm et al. (2024), we found that an overall reduction in the number of transitions between states may be crucial for explaining the complex brain functional organisation associated with anorexia. This result suggests a rigidity in brain connectivity that may characterise anorexia as an internalising mental disorder. Overall, analysing brain states helps us understand the underlying changes in dynamics and detect the functional brain alterations that reflect the psychological complexity of mental illness. These complexities may not be fully captured by more standard techniques (Peng et al. 2023).

## 5 Limitations

Several limitations should be acknowledged in this study. First, the small sample size may limit the statistical power of our analyses. For instance, a power analysis indicated that a sample of 42 participants (41 in this study) would be required to detect the association between time spent in State 3 and BMI with 80% power at *α* = 0.05. Additionally, controlling for the effects of comorbid conditions and medical treatments would have been ideal. Evidence suggests that different comorbidities have distinct functional connectivity profiles (Oathes et al. 2015) and their influence on our findings cannot be ruled out. However, our small sample size limited our ability to include these factors without risking overfitting. The cross‐sectional design of our study is another limitation, as it did not allow for the assessment of FC abnormalities in relation to changes in disease severity (Lotter et al. 2021). Furthermore, the correlation analyses between clinical data and dynamic brain state features were exploratory, intended to generate hypotheses for future research, and should therefore be interpreted with caution. For instance, the correlation between the EDI‐2 total score and time spent in specific brain states may not fully capture the nuances of individual subscales, potentially obscuring how specific aspects of eating pathology relate to brain dynamics. Moreover, due to adherence to our institutional practices, we used older versions of the EDI and DSM, which may limit the generalisability of the findings to current diagnostic frameworks. Finally, the technique used for dynamic analysis, the sliding window correlation method, is sensitive to its input parameters (Shakil et al. 2018; Yentes et al. 2013). Therefore, we suggest further research to explore the effects of different parameter settings and methodological approaches (Cabral et al. 2017; Eavani et al. 2013).

## 6 Conclusions

Our study indicates that patients with anorexia nervosa appear to be “stuck” in a specific functional brain configuration. This brain state may have clinical significance, as the time spent in it is associated with body mass index and eating disorder scores. A strong connection between the ventral attentional network and the somatomotor network is the primary characteristic of this state, which could help explain key symptoms of the disorder, such as reduced interoceptive awareness and distorted body image. Consistent with being ‘stuck’ in this state, we also found that the brain's ability to shift between different configurations is disrupted in anorexia nervosa. This disruption may reflect the cognitive inflexibility often observed in this disorder. However, it is important to note that comorbidities such as depression could play a significant role in these neural patterns. Future studies should aim to control for mood disorders and other comorbid conditions to more accurately isolate the contributions of anorexia nervosa to these brain configurations.

## Author Contributions

**Feliberto de la Cruz:** conceptualisation, methodology, data curation, visualisation, review and editing. **Monica Di Giuliano:** conceptualisation, methodology, data curation, formal analysis, visualisation, writing – original draft, and writing review and editing. **Andy Schumann:** review and editing. **Katrin Rieger:** data acquisition. **Karl‐Jürgen Bär:** review and editing.

## Conflicts of Interest

The authors declare no conflicts of interest.

## Supporting information

Supplementary Material

## Data Availability

The data that support the findings of this study are available from the corresponding author upon reasonable request.
