# Supplementary material for: Dynamic Functional Connectivity Markers in Anorexia Nervosa and Their Association With Clinical Symptoms: A Cross‐Sectional Study
Source: Eur Eat Disord Rev. 2025 Mar 10;33(4):835–45. doi: 10.1002/erv.3188 (PMC12171688; doi:10.1002/erv.3188)
Supplement: Supplementary file 1 — Supplementary Material [file ERV-33-835-s001.docx]

**Supplementary Materials**


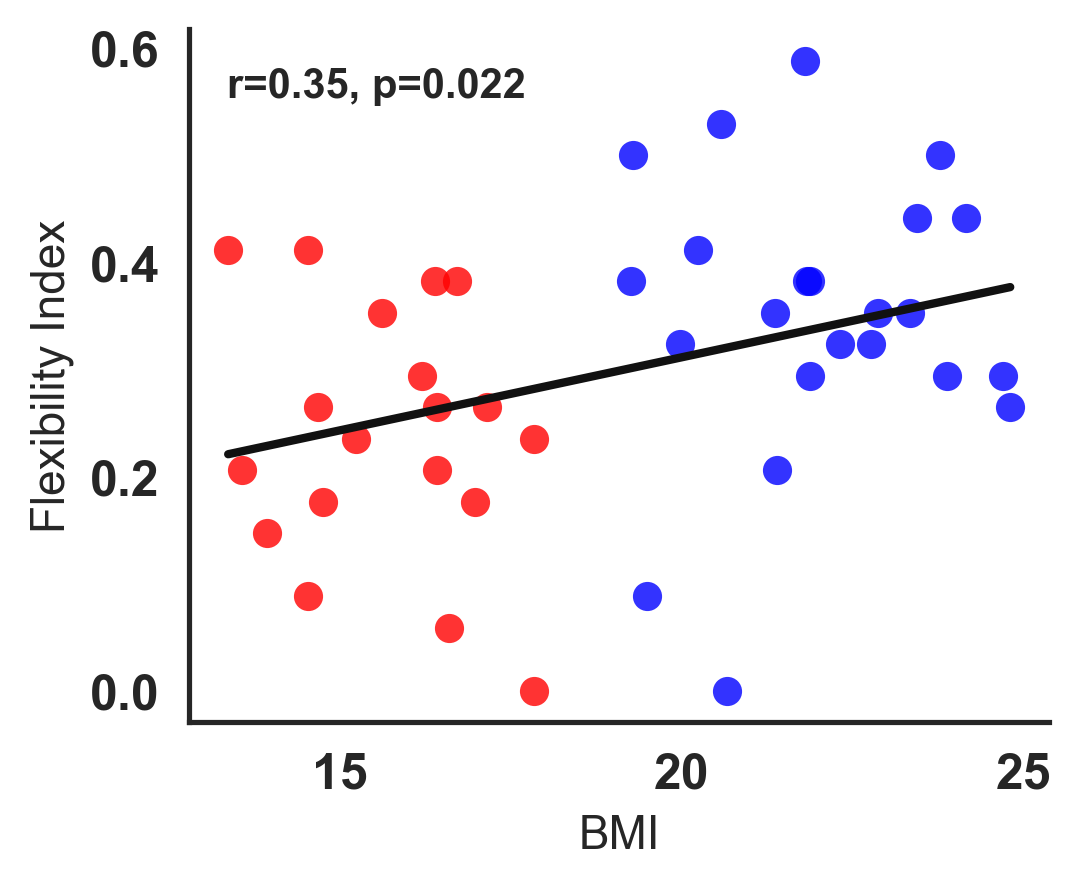

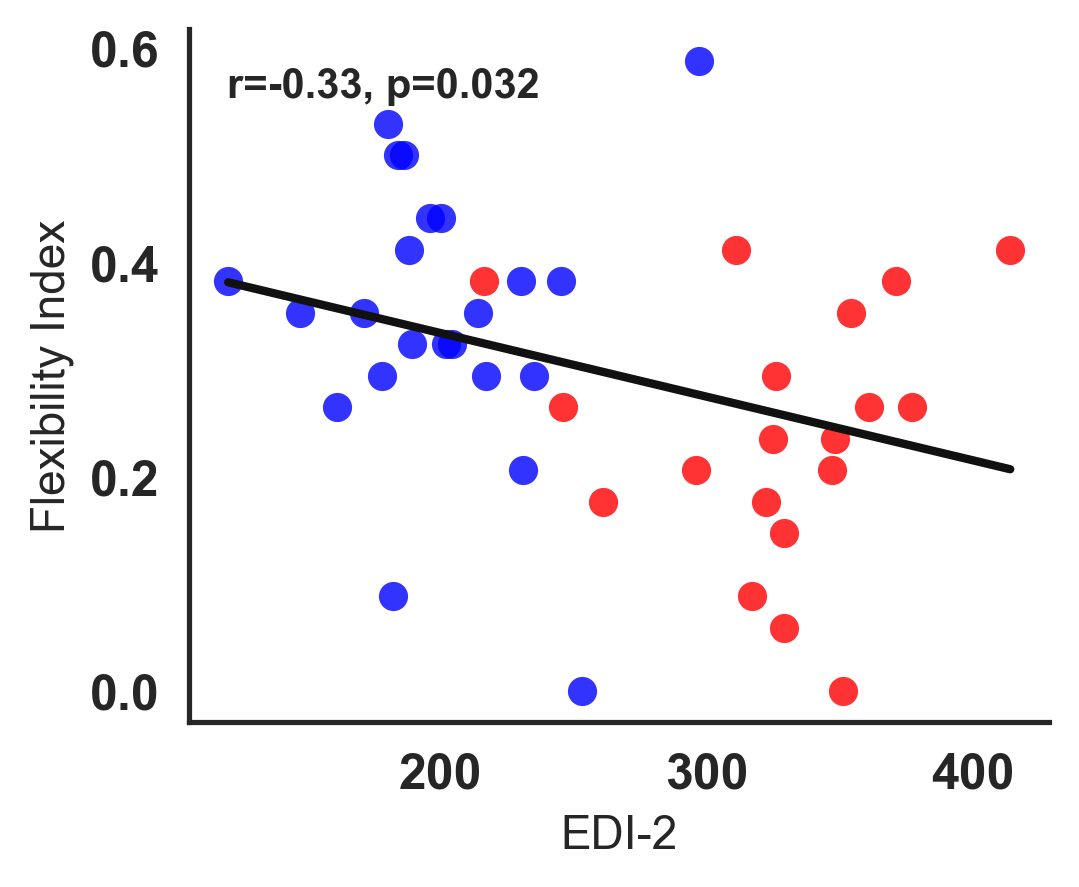


Figure S1: Association of the flexibility index with BMI (top) and EDI-2 (bottom). All p-values are uncorrected.


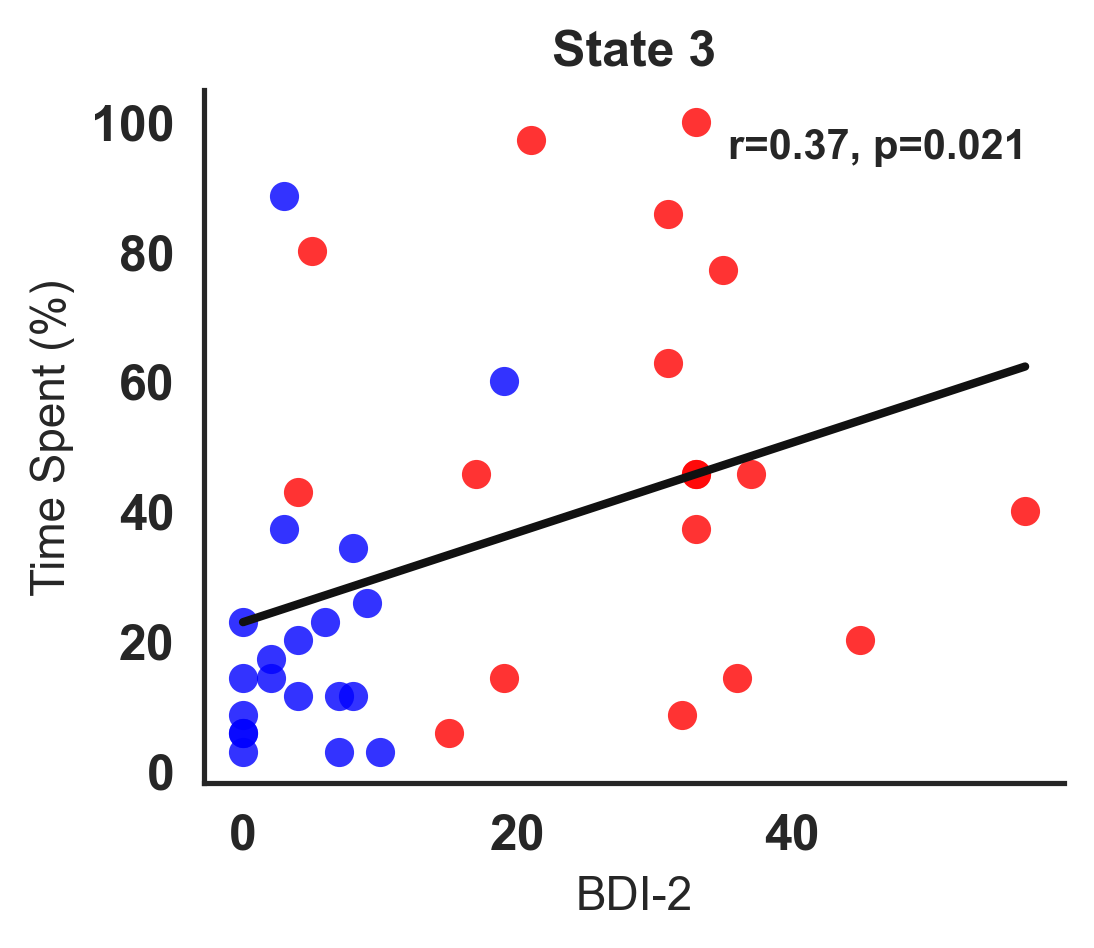

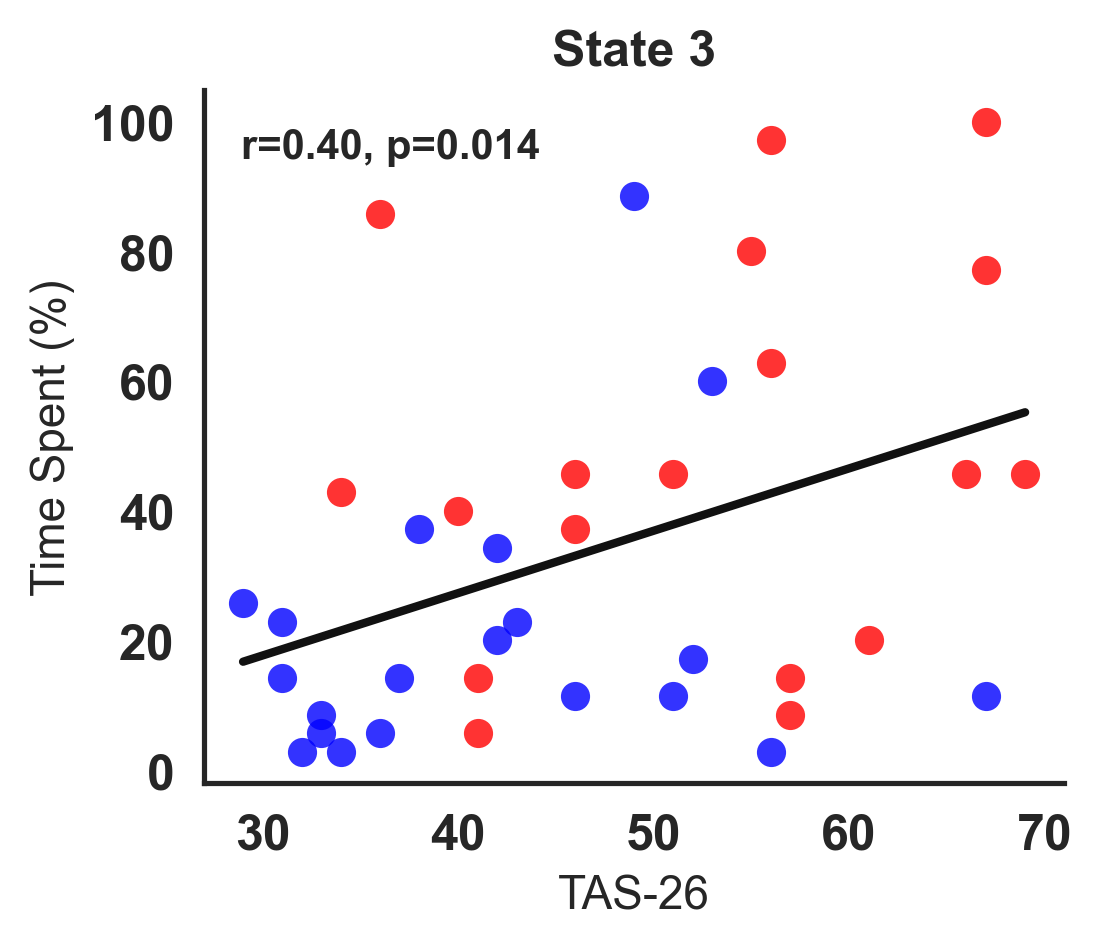


Figure S2: Association of the percentage of time spent in the clinically relevant brain state 3 with Beck Depression Inventory (BDI-2; top) and Toronto Alexithymia Scale (TAS-26; bottom). All p-values are uncorrected
